# Research governance in England needs improvements to ensure participant and site confidentiality in healthcare workforce research

**DOI:** 10.1186/s12961-026-01538-6

**Published:** 2026-09-28

**Authors:** Justin Aunger, Jane Ferguson, Esra Alkim Karaagac, Tommer Spence, Jill Maben, Ashok Patnaik, Russell Mannion, Yingxi Zhao, Nathan Proudlove, Helen Bodenham-Chilton, Kieran Walshe

**Affiliations:** 1 NIHR Midlands Patient Safety Research Collaboration, University of Birmingham, Birmingham, United Kingdom; 2 Department of Applied Health Sciences, College of Medicine and Health, University of Birmingham, Birmingham, United Kingdom; 3 Health Services Management Centre, School of Social Policy, University of Birmingham, Birmingham, United Kingdom; 4 School of Health Sciences, University of Surrey, Guildford, United Kingdom; 5 Nuffield Department of Medicine, University of Oxford, Oxford, United Kingdom; 6 Alliance Manchester Business School, University of Manchester, Manchester, United Kingdom

**Keywords:** Research governance, Governance, Healthcare, Workforce, Staff, Confidentiality, Bureaucracy, Anonymity

## Abstract

Sensitive healthcare workforce research relies on credible assurances of confidentiality for participating staff and organizations. Yet, National Health Service (NHS) research governance processes can compromise these assurances even before recruitment begins, particularly through processes such as site documentation, local principal investigator (PI) requirements, research passports, and portfolio reporting. These risks are especially important for studies examining organizationally sensitive topics such as bullying, speaking up, or patient safety issues, where perceived employer awareness may reduce participation or encourage self-censorship. From March 2026, the Health Research Authority introduced reforms for staff-only studies, including streamlined site set-up, reduced site visibility, and fewer requirements for local PIs or research passports in remote studies. However, further changes are needed to better balance transparency, oversight and confidentiality. Potential improvements include dedicated pathways for sensitive organizational research, restricted-access portfolio reporting, and more centralized governance documentation. Future changes should be co-designed with researchers and NHS research and development departments to protect confidentiality and improve research productivity, while maintaining ethical standards and accountability.

## The need for confidentiality in healthcare workforce research

Studies in National Health Service (NHS) settings that examine potentially sensitive organizational topics – such as mergers, bullying, fear of speaking up, implementation challenges, or patient safety issues – depend on a credible promise of confidentiality to both the participants taking part in the study and the host organizations involved [1, 2]. Without these assurances, sites and staff may refuse to participate or staff may not feel they can be honest and candid in what they say. Because site and participant identification are closely linked, a compromise of site confidentiality often creates risk for participant confidentiality too. For instance, once a site’s participation in a study becomes known, individual participants can become easier to identify, particularly where the research addresses a specific clinical area, role, or small department. However, NHS research governance arrangements – particularly the Integrated Research Application System (IRAS) and its site authorization requirements – contain structural features that can compromise confidentiality at both the site and participant levels before recruitment even begins.

This is a reflective commentary that draws on patterns we have observed while delivering multiple National Institute for Health and Care Research (NIHR)- and government-funded qualitative and mixed-methods studies of NHS workforce behaviour, organizational culture, and interorganizational collaboration, in roles spanning chief investigator, co-investigator and delivery researchers. Issues discussed are reflections only, and have not been systematically collected or quantified nationally or across studies, sites or research and development (R&D) departments.

This commentary also explores potential improvements, including some already being implemented by the Health Research Authority (HRA) in March 2026. IRAS and HRA approval operate on a United Kingdom-wide basis, with equivalent arrangements for Health and Social Care (HSC) organizations in Northern Ireland and for research bodies in Wales and Scotland. Because our own direct experience sits largely within NHS settings in England, the discussion below is framed primarily around the English system, although the same underlying architecture and many of the same confidentiality risks are shared across the devolved nations. Here, we focus primarily on workforce-focused research involving healthcare staff as participants, and in particular on studies addressing organizationally sensitive topics of the kind listed above, where the confidentiality risks we describe are most acute. Many of the same governance mechanisms apply to workforce research more broadly, but the consequences of a confidentiality failure are less severe where the subject matter is not organizationally sensitive.

Current research governance issues stem from a framework originally designed for patient-centred clinical trials that is poorly equipped for studies with a focus on the workforce. These research processes may compromise site and participant confidentiality through several avenues, outlined in Table 1. Concerns about the rigid nature of NHS research governance processes are widespread. For example, a recent survey of 252 health services researchers found that they are often perceived as disproportionate, inflexible and ill-suited to noninterventional and qualitative research [3]. The bureaucratic load also makes relatively “low risk” studies difficult to both deliver from a research perspective and reduces the likelihood for sites being willing to take part. Empirical work illustrates the scale and misalignment of these processes. For instance, Petrova and Barclay, in an audit of a low-risk NHS study, identified 89 individuals involved in research approvals and 491 exchanges outside formal systems such as IRAS, despite the study being considered “low-key” in terms of ethical risk [4]. Such findings demonstrate how governance systems designed for higher-risk clinical research may impose disproportionate administrative demands on qualitative and organizational studies, which incur a real-time (and thus monetary) cost.

**Table 1:** Example means by which confidentiality is compromised through current research governance processes

Governance process	How it undermines confidentiality	Aspect of confidentiality affected
Local information pack provision	IRAS form discloses all sites involved in the research to all involved sites (a national IRAS requirement under the pre-2026 process design; Table 2)	Site
Appointment of a local PI	At some sites, local PI may request to know who took part for safety or portfolio reporting purposes (a local interpretation we have encountered at some, though not all, sites)	Participant
Research passport	Research passports list all involved research sites, exposing sites to others (a feature of the national research passport system)	Site
NIHR Research Delivery Network portfolio reporting	Site identity exposed to others online with access to the Open Data Platform (a structural feature of the current national portfolio and Open Data Platform system)	Site
Delegation log	Names research staff and tasks, shared across organizations; colleagues of participants may be identifiable (the log itself is often a national requirement; sharing it across sites is a local implementation practice)	Participant/site

Each example is annotated to indicate whether it reflects a national requirement, a common local interpretation or a specific instance we have encountered in practice. NIHR, National Institute for Health and Care Research; PI, principle investigator

## Defining key terms

Throughout this commentary we use several related but distinct terms (Table 2). These distinctions matter because a given governance process may compromise site confidentiality without compromising participant confidentiality or vice versa. Please note these are our definitions, but are informed by definitions from the NIHR and *SAGE Encyclopedia of Communication Research Methods* [5, 6].

**Table 2:** Definitions of concepts discussed in this commentary

Term	Definition
Confidentiality	The responsibility and ability of the researchers to protect involved entities in a study from harm by keeping safe any personal or potentially identifying information
Anonymity	The extent to which a participant or site can or cannot be identified (not anonymous versus fully anonymous)
Transparency	The appropriate availability of information about a study’s authorization, conduct, governance and accountability, enabling scrutiny by relevant organizations and individuals
Site confidentiality	Nondisclosure about which specific organizations of study are participating in the research
Site visibility	The extent to which a participating site’s identity can be discovered by third parties – for example, via national systems such as the Open Data Platform (Table 1) – regardless of how carefully the research team themselves protect that information

## The process was primarily designed for clinical trials

Existing research governance processes originate in a system that treats the participant as a patient, the site as a treatment facility, and transparency as a safety mechanism. While valid for clinical trials, the principles are misaligned with workforce research where the organization itself is the subject of scrutiny, rather than just a facilitator of access to patients. That is not to say that transparency has no purpose in workforce research either. Transparency can help safeguard patients where staff behaviour has safety implications, and can reduce research waste by allowing scrutiny of how findings were reached. Confidentiality also matters in clinical research – for instance, in protecting patients from unwanted disclosure of their health information. The task is therefore not to trade confidentiality off against transparency and accountability, but to find governance mechanisms that can deliver transparency, accountability, participant protection, organizational duty of care, research integrity and public trust through means better suited to organizational rather than clinical research.

Until 2026, no dedicated IRAS pathway existed that could minimize site-to-site knowledge of study participation, and even the new changes to facilitate workforce research do not fully resolve the confidentiality risks created by local implementation, portfolio reporting or organizational awareness during recruitment. As such, confidentiality can get breached in a number of ways. For example, under current processes, distributing the local information pack (the full set of study documents including protocol, IRAS form, information sheets, etc.) to all sites included in the research exposes all included research sites to each other, potentially undermining the intended confidentiality protection. The research passport given by the NHS organizations to external researchers conducting the study on the site, which lists all involved organizations, raises the same confidentiality concerns.

Likewise, despite serving a very good purpose, the NIHR Research Delivery Network (RDN) portfolio can also compromise site confidentiality. The portfolio exists to track and support the delivery of research across NHS sites, enabling national oversight of study progress, recruitment performance and site activity. Inclusion on the portfolio is often a prerequisite for sites to access the infrastructure, resources and funding that the RDN provides, meaning that sites often decline to take part in a study if it is not on the portfolio. Therefore, researchers conducting workforce or organizational studies within the NHS are strongly incentivized to pursue portfolio adoption. Once listed, participating sites may become identifiable to users of the Open Data Platform (ODP) and other portfolio-linked public systems, through which some study information may be publicly visible [7]. This means that, even where a research team has taken care to protect site confidentiality in their communications and materials, the act of portfolio registration itself can expose an organization’s involvement to any interested party – including, potentially, the organization’s own leadership or staff. Currently, the research team can do portfolio reporting on behalf of all sites to ensure they receive payments. However, the ODP could use improvements to better maintain confidentiality.

A further example of the issue centres on the local principal investigator (PI) role. Historically, IRAS required each site to designate a named staff member as PI to oversee local study processes, including recruitment of participants, with the scope of this role often determined differently by local research and development (R&D) departments. In clinical studies, local PIs are generally clinicians, who manage patient recruitment and safety, and who need to know which patients are involved in the study to respond to adverse events. Many R&D departments still apply the most stringent interpretation of the rules to workforce-based studies, significantly increasing burden and timescales to set up studies. Thus, some sites require their local PIs to know who is taking part in the study (even with workforce-focused studies), which can create confidentiality concerns. This has a rationale rooted in transparency and safety for patient participants. However, in the case of research which focuses on staff, governance intended to protect participants and sites is instead the mechanism that undermines their confidentiality by revealing their participation, with no concomitant benefit to patients.

## How the confidentiality promise gets undermined

Participant information sheets for studies on sensitive topics typically inform staff that, for example, “your organization will not be identifiable in publications” or “your participation will not be identifiable to your organization”, and research ethics committees (RECs) approve these assurances in good faith. However, in the authors’ experience, some sites require sign-off from senior leadership before a study can be set up locally. Where this is required, site authorization may notify senior management about the project, its objectives and who is leading it locally. This may mean that people within the host organization know: (1) the study is taking place at their site, (2) what it is investigating, (3) who the local PI is and (4) the types of staff researchers are looking to recruit. This matters for data quality: if staff know or suspect their employer is aware a study is underway, it is a rational response to self-censor when speaking to researchers in an interview or when completing surveys. Research that requires a high degree of psychological safety, or that poses a reputational risk to organizations, such as research on unprofessional behaviour or patient safety, is particularly vulnerable because these are the topics on which staff are most likely to moderate their responses when under perceived institutional surveillance. The result may be under-reporting or more guarded qualitative accounts, limiting understanding of the very behaviours researchers aim to study [8].

A further problem is the gap between national approval and local implementation, which creates additional burden on researchers. HRA approval is intended to provide study-wide assurance on governance and legal compliance, including information governance, so that participating organizations should not need to duplicate checks already undertaken centrally. However, in practice, there is significant variation. Some local R&D departments still reopen questions about study design, documentation, recruitment routes, information governance or local risk, even when these have already been considered through HRA review, sponsor review or REC review. This concern has already been recognized in national guidance on information governance. A joint HRA, Clinical Research Network (CRN) and NHS R&D Forum statement from 2023 emphasizes that HRA approval provides study-wide assurances on data protection and information governance (IG) compliance and that participating organizations should not normally duplicate these checks locally [9]. However, issues are still present in 2026. This local variation is especially problematic for nonclinical workforce research because some R&D offices continue to interpret staff-only qualitative or survey studies through a clinical research lens.

Taken together, these mechanisms may compromise the confidentiality of involved sites and participants, potentially affect recruitment or data quality, and create administrative burden for both researchers and sites. Time spent navigating duplicated or disproportionate governance processes represents a direct cost to publicly funded research, diverting limited researcher and R&D capacity away from study delivery and analysis. It also negatively affects site onboarding. Supporting this, Snooks et al. found that some NHS Trusts decline to participate in workforce studies, citing critical capacity issues in their R&D units [3, 10]. For an R&D department under pressure, a low-risk interview or survey study about sensitive organizational issues may require many of the same local processes as more conventional clinical research while offering fewer obvious benefits: little or no treatment innovation, limited portfolio recruitment numbers, minimal local funding and potential reputational discomfort. The burden to R&D departments may therefore be disproportionate, which can create a disincentive for local participation.

## Towards workable solutions

Recent developments suggest that these concerns are beginning to be addressed. From March 2026, the Health Research Authority introduced a new governance pathway for studies involving NHS and social care staff as participants (Table 3) [11]. This process streamlines elements of site setup and reduces information-sharing requirements in IRAS, including limiting the visibility of participating sites and reducing the need for local site authorization in many cases. It also enables the chief investigator to act across sites without requiring local PIs and clarifies that research passports are not needed where studies are conducted remotely [11]. While not designed explicitly to address confidentiality concerns, this proportionate approach may reduce administrative burden and partially mitigate risks to site and participant confidentiality. Furthermore, a new system is currently being piloted to replace IRAS itself; it is called the Plan and Manage Health and Care Research system, which will be hosted on the gov.uk website. However, this new system may necessitate further training for site R&D departments as – in our experience with a pilot version – sites do not yet know how to process study modifications made using the new system.

**Table 3:** Proposed improvements from the HRA changes in March 2026 and the planned IRAS replacement system

Governance process	How the HRA changes in 2026 might help	How governance could go further to protect confidentiality
Local information pack provision	IRAS form no longer discloses all participating sites; in most cases, only the lead site is visible.	Remove necessity for a lead site as, in workforce studies, there is often no difference between “importance” of sites.
Appointment of a local PI	In most cases, the chief investigator can act as PI, overseeing the study remotely.	Remove expectation for local PIs entirely in low-risk staff studies; replace with a minimal “site contact” role without access to participant identities; introduce clear guidance preventing unnecessary access to participant-level data.
Research passport	Not required for studies conducted remotely (for example, online interviews or questionnaires); still required for research in NHS facilities and for letter of access.	Separate out the risk-checking functions of the passport from the list of study sites, or explicitly allow single-study or anonymized passports that do not list all sites (only the current relevant one); consider extending passport exemptions to all low-risk staff-only studies.
NIHR Research Delivery Network portfolio reporting	No current mitigation.	Allow pseudonymized or anonymized site listings on the Open Data Platform; introduce restricted-access tiers for sensitive studies; enable opt-out or delayed publication of site participation without loss of support or infrastructure access.
Delegation log	Held centrally by the chief investigator rather than shared across sites.	Standardize sponsor-held delegation logs across all studies; remove requirement for site-level sharing; ensure logs avoid linking identifiable staff to sensitive participation contexts unless strictly necessary.
Local R&D duplication and variable interpretation	The staff-participant route provides a reduced IRAS question set and proportionate guidance on capacity and capability.	Issue binding or strongly standardized guidance limiting local re-review of matters already assessed through HRA approval; create an escalation route where local requirements duplicate national review; improve training or policy dissemination from HRA to local R&D departments regarding staff-only, qualitative, survey and organizational research.

**Fig. 1:**
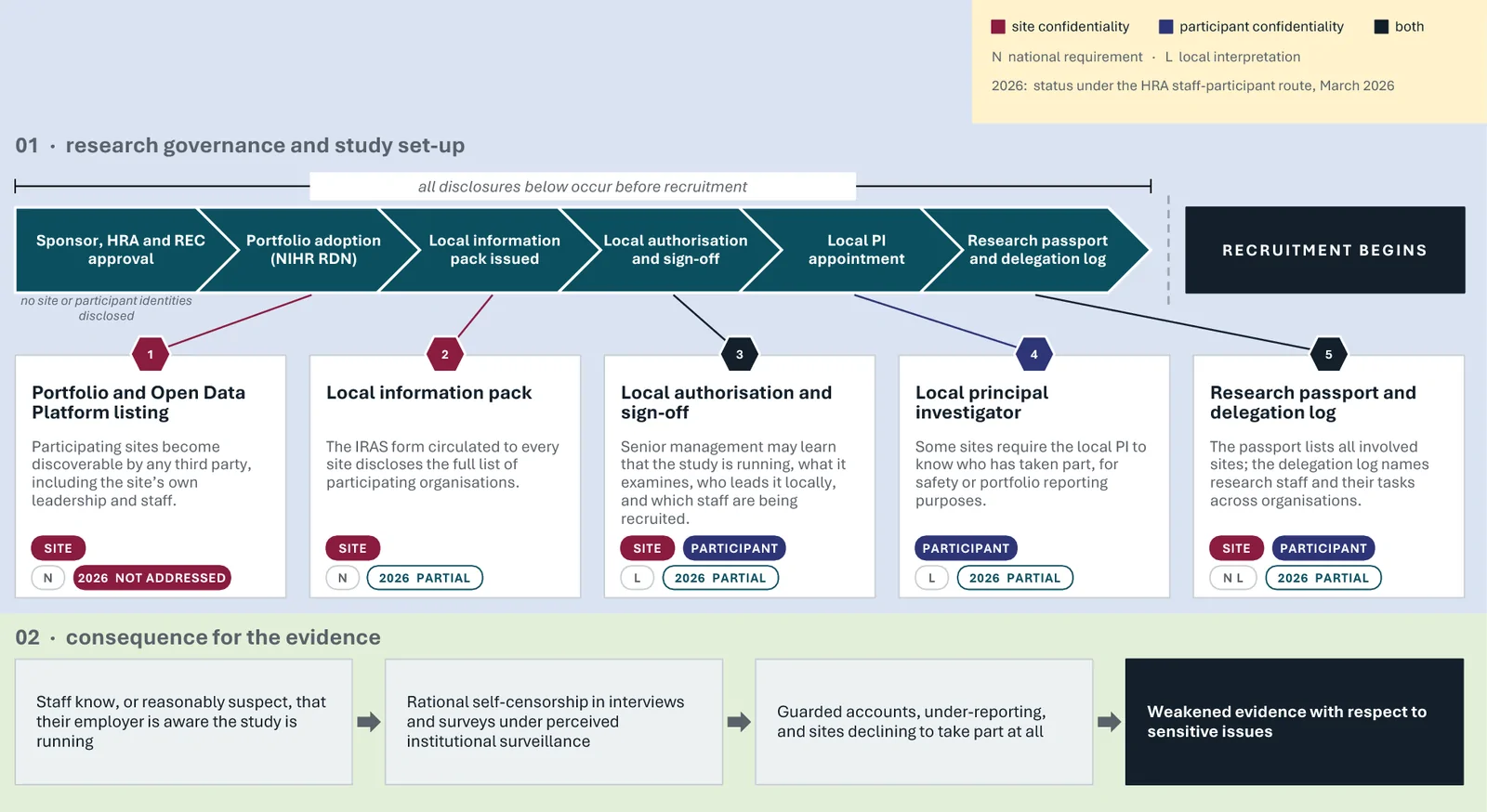
Overview of remaining confidentiality issues in research governance

This ongoing redesign through the HRA Research Systems Programme also aims to tailor application pathways to study type, which may improve alignment with workforce and organizational research. These developments sit within a wider push to reduce bureaucracy across the United Kingdom research system, including the 2022 Independent Review of Research Bureaucracy (the “Tickell Review”), which similarly identified unnecessary bureaucratic burden across research institutions and funders [12]. However, the HRA changes were being planned well in advance of this review, in as early as 2019, and thus do not originate with it. All these changes align closely with a broader direction of travel towards a more proportionate, streamlined approvals system. Figure 1 summarizes the remaining issues in research governance.

## Policy recommendations

Drawing on the issues and reforms discussed above, we set out six prioritized recommendations for further strengthening confidentiality protections in workforce-focused NHS and HSC research, without compromising accountability, participant safety or wider patient safety: (1) develop a dedicated HRA governance pathway for sensitive organizational research, extending the existing “validation-only” qualitative route; (2) remove or substantially reduce the requirement for local principal investigators in low-risk staff studies, replacing this with a minimal site-contact role that does not require access to participant identities; (3) issue binding national guidance that explicitly limits local re-review of matters already assessed through HRA approval, supported by a clear escalation route where this guidance is not followed (although this may lead to some additional centralized workload); (4) introduce pseudonymized, restricted-access or delayed-disclosure options for NIHR Research Delivery Network portfolio and Open Data Platform listings so that sites can access research infrastructure and funding without full public exposure of their participation; (5) waive local site sign-off entirely for low-risk workforce studies that have already been approved by the HRA and a research ethics committee, allowing sites to register directly with the HRA or research sponsor [10]; and (6) for future HRA governance reform, ensure co-design with researchers and NHS/HSC research and development departments, with confidentiality protection included explicitly, alongside administrative burden, as an evaluation criterion for future change.

### Ensuring safe and effective implementation

However, the proposed changes above are only suggestions. Proper implementation would need piloting, with evaluation of confidentiality, safety, participation and administrative burden. These national reforms can also only reduce confidentiality risks if they are implemented consistently by local R&D departments. A proportionate HRA pathway could be undermined if local offices continue to duplicate national review, require additional sign-off, or apply clinical-trial assumptions to staff-only organizational research. As such, the approach should be adopted at all levels of the system, in a manner proportionate to risk. Waiving local site sign-off would be appropriate only for clearly defined low-risk, staff-only studies that do not involve patient intervention, direct clinical contact or an evidenced need for local access to participant identities. Other studies should retain existing safeguards. Accountability should also be maintained where local PIs are removed, for instance, with the chief investigator and sponsor being accountable, while a named site contact could confirm local capacity and provide a route for urgent operational or safeguarding communication without access to participant identities. Similarly, portfolio pseudonymization should preserve secure, auditable mapping for authorized funders and oversight bodies, while limiting public visibility.

Appropriate site visibility also serves legitimate functions. For example, it can evidence that a study has an authorized host, support assessment of local capacity and capability, allow funders and oversight bodies to monitor delivery and the use of public resources, provide a route for urgent operational or safety escalation, and maintain public assurance that research is accountable [3, 4, 10]. These functions do not, however, require unrestricted public disclosure of participating sites or individual staff. The proposed approach would preserve them through secure central records, role-based access, audit trails, and a named site contact, while limiting disclosure to what is necessary.

## Conclusions

Recent developments from the HRA represent meaningful progress towards more proportionate governance. However, fundamental tensions remain between transparency, oversight and confidentiality in sensitive workforce research. Addressing these tensions will require governance processes that are explicitly designed for organizational research, rather than adapted from clinical trial frameworks. Future reforms should be co-designed with researchers and R&D departments to ensure that confidentiality protections are strengthened, without compromising accountability or wider participant safety. Without such changes, sensitive workforce research may remain vulnerable to delay, disclosure and site nonparticipation, with wider consequences for research productivity and the efficient use of valuable public funding.

## Data Availability

No datasets were generated or analysed during the current study.
